# Measuring and modelling the effects of systematic non-adherence to mass drug administration

**DOI:** 10.1016/j.epidem.2017.02.002

**Published:** 2017-03

**Authors:** Louise Dyson, Wilma A. Stolk, Sam H. Farrell, T. Déirdre Hollingsworth

**Affiliations:** a Mathematics Institute, University of Warwick, Coventry, UK; b School of Life Sciences, University of Warwick, Coventry, UK; c Department of Public Health, Erasmus MC, University Medical Center Rotterdam, Rotterdam, The Netherlands; d London Centre for Neglected Tropical Disease Research, Department of Infectious Disease Epidemiology, St Mary's Campus, Imperial College London, London WC2 1PG, UK

**Keywords:** Neglected tropical diseases, Coverage, Systematic non-compliance, Systematic, non-adherence, Modelling

## Abstract

It is well understood that the success or failure of a mass drug administration campaign critically depends on the level of coverage achieved. To that end coverage levels are often closely scrutinised during campaigns and the response to underperforming campaigns is to attempt to improve coverage. Modelling work has indicated, however, that the quality of the coverage achieved may also have a significant impact on the outcome. If the coverage achieved is likely to miss similar people every round then this can have a serious detrimental effect on the campaign outcome. We begin by reviewing the current modelling descriptions of this effect and introduce a new modelling framework that can be used to simulate a given level of systematic non-adherence. We formalise the likelihood that people may miss several rounds of treatment using the correlation in the attendance of different rounds. Using two very simplified models of the infection of helminths and non-helminths, respectively, we demonstrate that the modelling description used and the correlation included between treatment rounds can have a profound effect on the time to elimination of disease in a population. It is therefore clear that more detailed coverage data is required to accurately predict the time to disease elimination. We review published coverage data in which individuals are asked how many previous rounds they have attended, and show how this information may be used to assess the level of systematic non-adherence. We note that while the coverages in the data found range from 40.5% to 95.5%, still the correlations found lie in a fairly narrow range (between 0.2806 and 0.5351). This indicates that the level of systematic non-adherence may be similar even in data from different years, countries, diseases and administered drugs.

## Highlights

• We review models of systematic non-adherence and propose a new model for the effect.
• We use two simplified models to explore the effects of systematic non-adherence.
• We find that systematicness has a significant impact on the campaign outcome.
• The number of rounds attended can be analysed to find the level of systematicness.
• In published data the correlation between treatment rounds is between 0.281and 0.535.

## 1 Background

Mass drug administration (MDA) is the cornerstone of a number of control programs, particularly helminth control and trachoma programs, and also forms a part of the suite of interventions for diseases such as malaria and yaws (World Health Organization, 2013). These programs are based on the use of drugs with a good safety profile which can be distributed without close clinical supervision, and are usually prioritised because they are much more cost-effective than screening and treating only infected individuals due to the logistic costs involved (Brooker et al., 2008, Holland et al., 1996). For neglected tropical diseases (NTDs), billions of individuals have been treated in MDA programs. In some of these programmes key disease control goals have been met so that MDA could be stopped (e.g. MDA programmes for lymphatic filariasis in Egypt, Yemen, Sri Lanka, etc. World Health Organization, 2015). However, other programs are not achieving the expected goals, and so we are facing the question of why these “failures” are occurring and how better to measure the effectiveness of control programs.

Mathematical modelling plays an important role in the design of MDA programs—who to treat, when to treat (Anderson et al., 2012, Anderson et al., 2015, Coffeng et al., 2014, Coffeng et al., 2015, Gambhir and Pinsent, 2015, Gurarie et al., 2015, Irvine et al., 2015, Jambulingam et al., 2016, Liu et al., 2015, Singh and Michael, 2015, Stolk et al., 2015, Truscott et al., 2015, Winnen et al., 2002)—and in setting the ‘expected’ prevalence after a certain number of rounds, particularly for onchocerciasis (Tekle et al., 2016). Modelling studies have highlighted the importance of coverage (the proportion of the target population who are treated), with high coverage leading to more rapid declines in prevalence and sustained high coverage leading to the possibility of elimination (Okell et al., 2011, Slater et al., 2014). Empirical studies (Krentel et al., 2013, Brieger et al., 2012, King et al., 2011, Boyd et al., 2010) have highlighted that some individuals do not receive treatment not through chance, but through a systematic lack of access to the treatments (such as workers who are away during the daytime treatments, Rock et al., 2015, Mpanya et al., 2012) or lack of acceptance of the treatment. These studies, among others, investigate how treatment campaigns and interventions are affected by the cultural and socio-economic contexts in which they occur (Krentel et al., 2016, Parker and Allen, 2013a, Parker and Allen, 2013b, Roy et al., 2013, Shuford et al., 2016). In addition, many investigations into treatment campaign coverage highlight the unreliability of reported coverage data, further complicating modelling efforts (Brieger et al., 2011, Cromwell et al., 2009).

Early modelling work for lymphatic filariasis highlighted how these types of systematic non-adherence to a program can undermine the success of that program and, depending on the size of the untreated group, act as an important reservoir for infection, leading to onward transmission to the rest of the population (Plaisier et al., 2000). The decision to proceed with post treatment surveillance may be based on the reported coverage levels combined with modelling predictions (for example in lymphatic filariasis, where achieving around 7 years of high coverage is seen as a trigger to begin transmission assessment surveys). It is important to measure and understand these effects to prevent the danger of stopping too soon or continuing costly interventions after they are no longer needed. If untreated individuals are geographically clustered, then this type of non-adherence, or lack of access, can lead to hotspots of ongoing transmission. A more recent study applied the method by Plaisier et al. (2000) (which was previously used in a deterministic setting) to study the effect of different models of systematic non-adherence in an individual-based model of helminth infections (Farrell et al., 2017).

Different modelling groups have approached modelling systematic non-adherence (which we shall use as a catch-all term for the situation when some parts of the population repeatedly do not receive treatments) in different ways, but these different methods have never been explicitly compared with respect to the resulting simulated coverage patterns or the resulting predicted trends in infection. Here we aim to formalise a new model for this behaviour which is flexible enough to capture the different methodologies and allow more direct comparison with empirical data. We investigate the impact of different assumptions for systematic non-adherence using a simple susceptible-infected-susceptible (SIS) model and a helminth model. We use examples from the small number of published empirical studies which measure these phenomena to evaluate the size of the effect, and discuss the value of further surveys to inform future modelling work. We note that our work is an attempt to capture effects that may be general across multiple different diseases and to apply this to any particular disease or country would require more in-depth study of the specific situation.

## 2 Overview

We will begin by reviewing how various models include systematic non-adherence and introducing a new way of modelling treatment that allows the user to specify the level of systematic non-adherence in addition to the coverage (Section 3). Then we will consider the consequences of systematic non-adherence in MDA campaigns by implementing the various schemes into a (very simplified) model of SIS dynamics and one for helminth infections, demonstrating that the level of systematic non-adherence has a significant impact on the outcome of interventions (Section 4). Finally, we will consider what data is required (and how to analyse it) to assess the level of systematic non-adherence and will show that for the limited data in the literature the correlation between rounds of treatment lies in a narrow range of values (Section 5).

## 3 Modelling descriptions of systematic non-adherence

Many modelling descriptions of systematic non-adherence have been used in a variety of models of different diseases. Here we review and compare the different schemes and propose a new method.

**Fig. 1:**
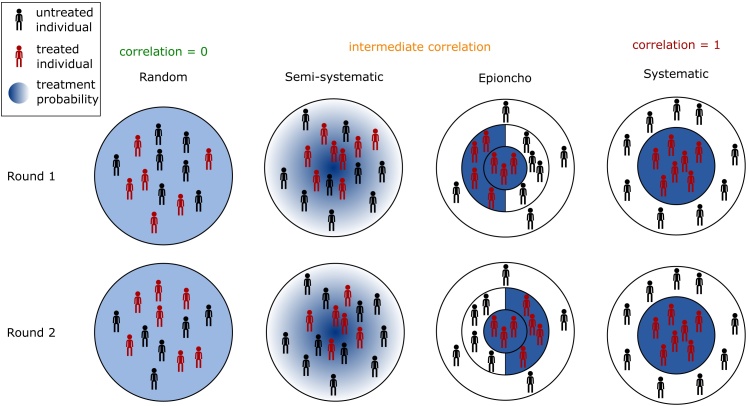
A schematic to represent the different schemes used to model treatment campaigns. For each scheme we give two rounds of treatment. Individuals receiving treatment in that round are coloured red, whereas those not receiving treatment are in black. In each diagram the background colour represents the probability that a person will receive treatment in that round, from white (never receive treatment) to dark blue (always receive treatment). The controlled correlation scheme is not shown explicitly in this diagram but can give different levels of systematicness depending on the correlation parameter used.

### 3.1 List of schemes

1. Random – each round a randomly selected group of individuals are treated. (1 parameter – coverage)
2. Population partitioning:
  (a) Fully systematic – two groups that are treated: every round; or never treated (1 parameter – coverage)
  (b) Deterministic approximation to a semi-systematic scheme (number of parameters depends on the scheme)
3. Semi-systematic – each individual has a probability *p*_*i*_ (the same for every round) of being treated in each round. (1 parameter – coverage)
4. Variable correlation scheme – treated individuals are distributed with a given expectation while correlation is controlled by a given parameter. (2 parameters – coverage and correlation)
  (a) Scheme by Griffin et al. (2010) and Irvine et al. (2015)
  (b) Controlled correlation scheme introduced in this paper

We discuss each scheme in detail below.

#### 3.1.1 Random

**Fig. 2:**
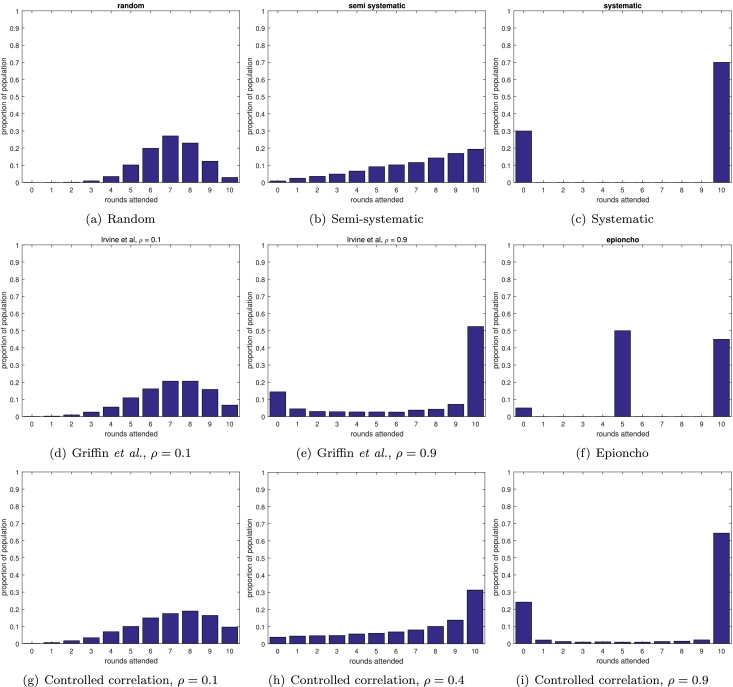
Distribution of then number of rounds of treatment experienced by the population for different schemes at 70% coverage.

The majority of modelling predictions for the outcome of mass drug administration campaigns assume random coverage (Truscott et al., 2015, Gambhir and Pinsent, 2015, Liu et al., 2015, Blok et al., 2015, Pandey et al., 2015, Singh and Michael, 2015, Gurarie et al., 2015, Anderson et al., 2015). In this scheme, each individual in each round has the same probability, *c*, of receiving treatment, where *c* is the coverage achieved by the campaign. If the campaign continues running for enough rounds then eventually all individuals will have received at least one treatment. Since each individual has the same probability of being treated in each round, the proportion of the population that is never treated drops off very quickly as the number of rounds increases. To ensure a probability of at most *T* that a randomly selected individual has never received treatment, at a given coverage *c*, requires greater than log(*T*)/log(1 − *c*) rounds of MDA. The distribution of number of rounds attended in the population after 10 rounds at 70% coverage is shown in Fig. 2(a), demonstrating that the proportion of the population that have never attended a round is very small. The distribution is clustered around 7 rounds attended, since this would be the mean number of rounds attended after 10 rounds at 70% coverage under this scheme.

#### 3.1.2 Population partitioning

A simple way of incorporating systematic non-adherence into any model (deterministic or individual-based) is to partition the population into subpopulations that receive different treatment regimes.

The most extreme version is a fully systematic scheme, where every individual either attends every round, or never attends any rounds. This scheme only requires knowledge of the coverage, which gives the proportion of the population that attends every round. This scheme is most useful as a ‘worst case scenario’. This scheme is implemented as one of multiple schemes in a model for lymphatic filariasis (LYMFASIM: Stolk et al., 2008, Stolk et al., 2003, Plaisier et al., 1998), and is also studied in a deterministic model for onchocerciasis (Turner et al., 2015, Turner et al., 2014a, Turner et al., 2014b). Plaisier et al. (2000) assessed the comparative effectiveness of MDA for lymphatic filariasis with random, systematic or semi-systematic coverage schemes. The scheme is shown in Fig. 2(c).

Another partition would first assume a subpopulation that never attends screening and then use another model of choice for the remaining population. For example, incorporating a randomly-participating population and a never-participating population (HAT: Rock et al., 2015, hookworm: WORMSIM: Coffeng et al., 2015) or a never-par-ticipating population and a semi-systematically participating population (see Section 3.1.3) (onchocerciasis: ONCHOSIM: Plaisier et al., 1990). To approximate a semi-systematic scheme in a deterministic model, one can partition the population into groups that receive treatment at different rates. For example, a PDE model of onchocerciasis (EPIONCHO: Basá nez and Boussinesq, 1999, Turner et al., 2013) the authors split the population into four groups: one in which individuals participate every round; one where they participate in even rounds; one participating in odd rounds; and one group that never participates. This scheme is very distinctive when we consider the number of rounds attended by the different populations (Fig. 2(f)), and it would be very surprising if this was seen in real data. However it is important to remember that this scheme is not intended as a direct representation of the real world, but as an attempt to make a semi-systematic scheme in a deterministic setting. In addition, this scheme could be extended by adding further subgroups that are treated every 1, 2, 3, … rounds or indeed including a separate subpopulation for each possible combination of rounds attended.

#### 3.1.3 Semi-systematic

Under the semi-systematic scheme the *i*th individual has a probability *p*_*i*_ of attending a round of treatment. To achieve a coverage *c*, each individual must have probability $p_{i}=u_{i}^{(1-c)/c}$, where *u*_*i*_ is a uniformly distributed random number on the interval [0, 1]. Note that this scheme differs from the random scheme, since the probability differs between individuals (but is the same for each round), whereas in the random scheme the probability is the same for all individuals (and is also the same for all rounds). This can be extended to include sex- and age-related participation rates. The difference between the semi-systematic scheme and the random scheme may be easily seen in Fig. 2(b) where it is clear that the semi-systematic scheme results in a larger proportion of the population receiving zero or very few rounds of treatment, even at 70% coverage levels, thus having the potential to seriously undermine MDA campaigns. The semi-systematic scheme has been considered in models of lymphatic filariasis (LYMFASIM: Jambulingam et al., 2016, Plaisier et al., 1998, Plaisier et al., 2000, Stolk et al., 2003, Subramanian et al., 2004), hookworm (WORMSIM: Coffeng et al., 2015), onchocerciasis (ONCHOSIM: Coffeng et al., 2014, Plaisier et al., 1990, Stolk et al., 2015), and schistosomiasis (SCHISTOSIM: de Vlas et al., 1996).

#### 3.1.4 Variable correlation schemes

It is possible to fit many of the preceding schemes into a general framework in which the correlation between rounds attended (*i.e.* if an individual attends one round to what extent they are more likely to attend others) is set by the user in addition to setting the coverage achieved. This was first attempted by Griffin et al. (2010) and their scheme was subsequently used by Irvine et al. (2015) (details in the supplementary information). However, while their scheme gives a way of increasing the correlation between rounds, it does not allow the user to directly set the correlation exactly. In addition, there is no way of reproducing the semi-systematic scheme described above, since higher correlations are achieved by including a larger number of people that always or never attend treatment (see Fig. 2(d) and (e)).

We propose a new scheme (using a method by Qaqish, 2003) in which both the coverage, *c*, and the correlation between rounds, *ρ*, may be controlled exactly. We call this scheme the controlled correlation scheme. The procedure is as follows: in the first round, each person attends treatment with probability *c*. In round *k*, individual *i* attends treatment with probability (*c*(1 − *ρ*) + *ρR*_*i*_)/(1 + (*k* − 2)*ρ*), where *R*_*i*_ is the number of rounds attended by person *i* so far. It is clear that the more rounds a person has previously attended, the more likely they are to attend subsequent rounds, and the strength of this effect is controlled by *ρ*. If *ρ* = 0 then this reduces to the random scheme (Section 3.1.1, Fig. 2(a) and (g)), and if *ρ* = 1 then each person will attend round *k* if, and only if, they attended the first round, thus reducing to the systematic scheme in Section 3.1.2 (Fig. 2(c) and (i)). In fact this scheme is equivalent to giving each person a parameter that gives their probability of attending any round (which is fixed for that person), as in the semi-systematic scheme, but drawing that parameter from a Beta distribution with parameters *α* = *μ*_*y*_(1 − *ρ*)/*ρ*) and *β* = (1 − *μ*_*y*_)(1 − *ρ*)/*ρ* (see supplementary information).

As for previous schemes, the variable correlation scheme may be straightforwardly applied to subpopulations with different attendance parameters (for example different age groups) by generating attendances separately for each subpopulation. It is also possible to extend this scheme (see supplementary information) to include additional correlated variables to model correlations between adherence to different types of interventions or between risk and adherence to interventions. For example, it might be that people who are likely to receive drug treatments are also more likely to receive indoor residual spraying (IRS) or to receive and use bednets (Griffin et al., 2010).

## 4 What are the consequences of systematic non-adherence?

To assess the impact of the schemes discussed in Section 3.1, we use two very simplified models of infection dynamics: an ‘SIS’ model; and a simplified helminth infection model, before briefly considering the effect of correlations between treatment and infection risk.

### 4.1 SIS dynamics

**Fig. 3:**
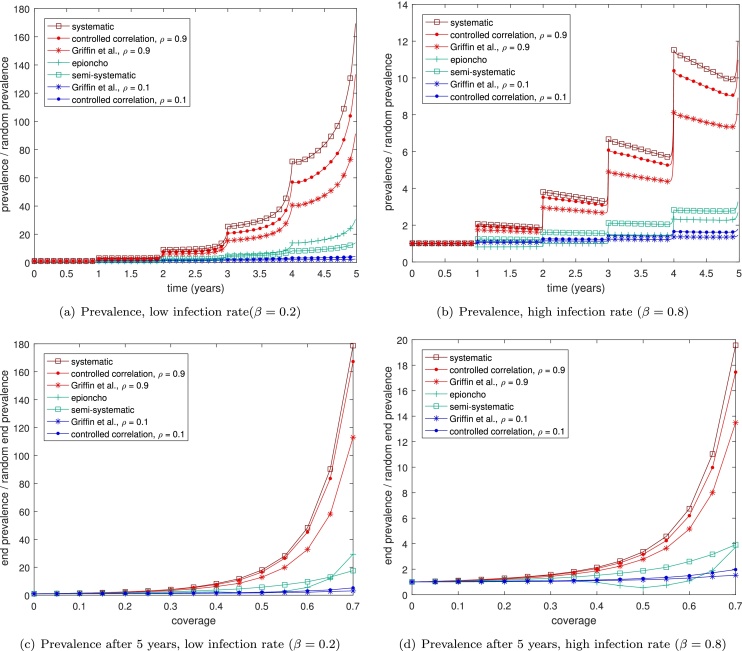
The impact of different types mass drug administration coverage on: (a) and (b) the prevalence; (c) and (d) the prevalence after 5 years; of an SIS model over multiple rounds of treatment, for different infection rates, *β* when using a coverage of 70%. In each plot the schemes we expect to have high systematic non-coverage are shown in red, those that are more random are in blue, and those with some systematicness are shown in green. We take the rate of recovery, *γ* = 0.15. Lines are averaged over 1000 simulations and are scaled by the prevalence attained when using the random coverage scheme. For reference, the random coverage scheme attains a prevalence 0.0003 for *β* = 0.2 and of 0.03 for *β* = 0.25.

In our SIS model individuals are either infected (*I*) or susceptible (*S*), and infecteds infect susceptibles at a rate, *β* and recover at a rate *γ*. We simulate the system stochastically using the Gillespie algorithm (Gillespie, 1977). In addition, we include mass treatment events using the schemes in Section 3.1. We consider two different model outputs: the prevalence of infection over time; and the prevalence of infection after 5 years. For each prevalence measure, we give the prevalence scaled by the prevalence achieved by most effective scheme: a fully random treatment campaign. For example, in Fig. 3(a) we see that the prevalence after 5 years can be up to 180 times greater for a systematic scheme than for random coverage.

#### 4.1.1 Impact of the intervention

We run the model to steady state (200 years, giving a starting prevalence of 0.08 for *β* = 0.2 and 0.25 for *β* = 0.8) before beginning the plotted simulations with a mass drug treatment at year zero. Code for the simulations may be found as supplementary information. At the second round the different schemes will have a different level of overlap with previously cured individuals. The more ‘systematic’ schemes will tend to re-treat individuals who were previously treated at time zero, so that this will only decrease the prevalence if those individuals have since been reinfected. Over repeated treatments, the difference between the more and less systematic schemes becomes progressively greater (Fig. 3 (a) and (b)). Varying the coverage levels and considering the prevalence after 5 years demonstrates that the effect of systematic non-adherence is greater at higher coverages

We may also investigate different endemic settings, in which infection happens at different rates. Systematic non-adherence has a much greater effect when infection rates are slower (Fig. 3(a) and (b)), since at lower infection rates the individuals that are repeatedly treated in the more systematic schemes are unlikely to have become reinfected between treatments. At the extreme, if the infection rate is so high that all individuals are reinfected by the end of a year, it is clear that the different schemes would have exactly the same impact, since the coverage is the same in all the schemes.

#### 4.1.2 Prevalence after 5 years

To investigate more how the different schemes vary with coverage rates, we consider the prevalence after 5 years for varying levels of coverage and different infection rates (Fig. 3(c) and (d)). These figures display an even more clear distinction between the different schemes, with more systematic schemes displaying huge differences in prevalences. The effect of systematic non-adherence is more pronounced at higher coverages, since the difference between the populations treated is greater when more people are being treated in general. At very high coverages the less systematic schemes can eliminate the disease from the population, and for this reason we do not give data for greater than 70% coverage (since we scale by the prevalence from the random scheme, which is often zero after 5 years at high coverages).

### 4.2 Helminth dynamics

The impact of systematically re-treating individuals is less clear in a model of helminth infections, since individuals are not regarded to be simply infected or susceptible. Instead they are infected with a number of worms (which may be zero). In this model the prevalence of the disease in the population is given by the proportion of the population that have a non-zero number of worms. When individuals are treated they are not necessarily fully cured, but instead a proportion of their worms are killed. In these models, therefore, individuals that are treated multiple times are more likely to be cured than those that only receive one treatment. Hence it is possible that a degree of 'systematicness’ could reduce the prevalence in the population, particularly at low coverages, by concentrating those treatments so that a lower subpopulation is treated, but they are more likely to be fully cured.

We again take a very simplified model to highlight the differences in the treatment schemes without including much detail about the infection dynamics. In particular, we are not modelling any particular type of helminth, and the parameters we use are not informed by real world data. We do not include any details of worm replication which in reality, depending on the species, can be sexual or asexual, and we only consider adult worms, neglecting larvae stages and vectors of infection, such as insects or snails. Instead we use a model in which individuals are infected with a number of worms, which die at a rate *γ*. An individual *i* gains worms increase through contact with another infected individual, *j*, at a rate (*β*∑_*j*_*W*_*j*_/*N*)*C*/(*C* + *W*_*i*_), where *β* is the infectivity, *N* is the population size and *C* gives density dependence, so that as the number of worms in a single individual increases, the ‘space’ for new worms decreases. We also include death of the individual, which is paired with new births so that the net effect of a person dying is that they are replaced by a completely uninfected person.

#### 4.2.1 Plotting the prevalence during a mass drug campaign

**Fig. 4:**
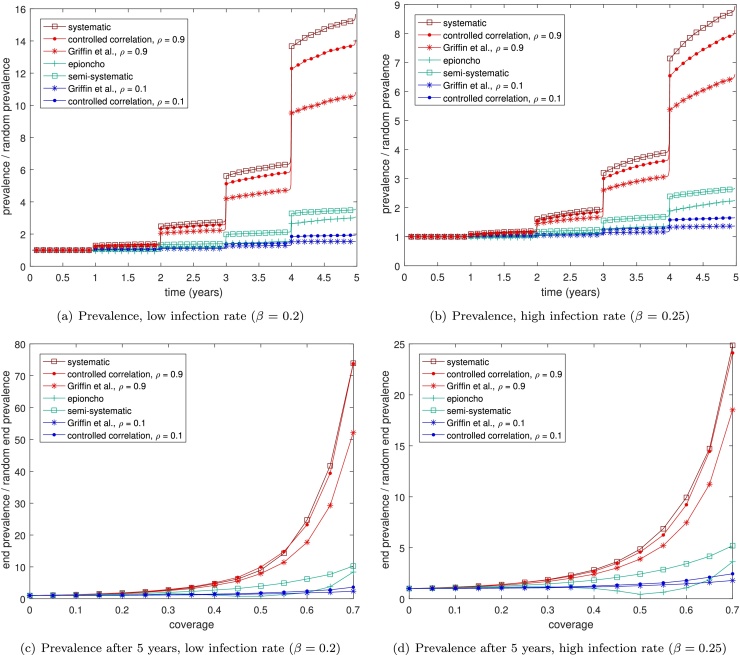
The impact of different types mass drug administration coverage on: (a) and (b) the prevalence; (c) and (d) the prevalence after 5 years; of a simplified helminth model over multiple rounds of treatment, for different infection rates, *β* when using a coverage of 70%. In each plot the schemes we expect to have high systematic non-coverage are shown in red, those that are more random are in blue, and those with some systematicness are shown in green. We take the rate of death of worms to be *γ* = 0.1, the birth/death rate of people to be 0.1, the density dependence parameter to be *C* = 50 and assume that each treatment kills 70% of that person's worms. Lines are averaged over 1000 simulations and are scaled by the prevalence attained when using the random coverage scheme. For reference, the random coverage scheme attains a prevalence 0.0005 for *β* = 0.2 and of 0.016 for *β* = 0.8.

As before we plot the prevalence in the population over time during a mass drug campaign, scaled by that attained by a random coverage model. We run the model to steady state (200 years, giving a starting prevalence of 0.15 for *β* = 0.2 and 0.25 for *β* = 0.25) before administering a treatment round at time = 0 years (Fig. 4). Code for the simulations may be found as supplementary information. We previously mentioned the possibility that concentrating treatments in a subpopulation may lead to a lower prevalence (*i.e.* proportion of the population that is infected) while still increasing the average number of worms. We note here, however, that this is never observed in our model simulations. An increase in ‘systematicness’ always leads to higher prevalences in our model simulations (Fig. 4), as was observed in the SIS system. As in the SIS model, the effect of systematic non-adherence is more pronounced at low infection rates (Fig. 4(a) and (c)). We note that the effect is somewhat reduced compared to the SIS model with systematic treatment producing prevalences up to 70 times that for random treatment in the helminth model, compared to 180 times in the SIS model. However this may be influenced by the parameter values chosen.

### 4.3 Correlations between treatment and infection risk

Another type of systematic effect that can have a large influence on the system dynamics is a correlation between adherence and infection risk. In this situation individuals that are unlikely to be treated also have a higher risk of being infected. We would expect this to have negative consequences for a treatment campaign, since the population that is most likely to be infected is also the least likely to be treated for that infection.

We may study this using a very simple model, in which each individual *i* has some probability *T*_*i*_ of receiving treatment, and acquires disease at some rate *β*_*i*_, then their probability *P*_*i*_(*t*) of being infected at time *t* is given by

(1) $$\frac{dP_{i}}{\mathrm{d t}}=\beta_{i}(1-P_{i})-T_{i}P_{i},$$

(2) $$P_{i}(T_{i},\beta_{i},t)=\frac{\beta_{i}+T_{i}e^{-t(T_{i}+\beta_{i})}}{\beta_{i}+T_{i}}.$$

We find by studying this system (see supplementary information) that a positive correlation between treatment and infection is likely to initially increase prevalence compared to a situation without such a correlation. This can be intuitively understood, since at the beginning of a treatment campaign it is better to focus on the ‘easy gains’ by treating those people that will not quickly become reinfected. This effect is seen in simulations of our SIS and helminth models incorporating correlations between infection risk and treatment (see supplementary information).

## 5 Using data to assess the extent of systematic non-adherence

The preceding sections have demonstrated the impact of systematic non-adherence on the prevalence of disease. In addition, the form of the non-adherence also has an impact on elimination time and disease burden over time. While the coverage is generally acknowledged to have a fundamental impact on the success of a campaign, the form that coverage might take is less widely studied. For this reason good quality data on the level and form of non-adherence is relatively sparse. It is important to note, however, that even if the coverage and correlations are known, this does not fully specify the distribution of attendance. In spite of this, we will argue that data about non-adherence should be routinely collected during a mass drug administration campaign, in the same way that data about coverage is commonly taken and studied. This would represent a significant step forward in quantifying systematic non-adherence.

### 5.1 Existing data

For helminth infections, a systematic review was undertaken by Shuford et al. (2016). Many of the studies included in this review reported coverage data, or were investigations into the reasons for non-compliance. These papers give insight into factors associated with non-compliance, but not the extent to which an individual is likely to receive multiple rounds of treatment. Discovering the reasons for non-compliance is invaluable when attempting to increase coverage, but for modelling purposes a more simple measure of the level of correlation between treatment rounds would significantly increase the accuracy of predictions. Some published articles (King et al., 2011, Brieger et al., 2012) hint at access to data that would give this information, but correlation measures are not generally calculated or published. A few articles do include data of the form plotted in Fig. 2 (Newell, 1997, Plaisier et al., 2000, Brieger et al., 2011, Mathieu et al., 2006, El-Setouhy et al., 2007). Notably Plaisier et al. (2000) also include a comparison of the distribution of rounds attended against random, systematic and semi-systematic attendance, and conclude that semi-systematic attendance is the most realistic of the three schemes. Since numerical data is not given in Plaisier et al. (2000), we will consider only Newell (1997), Brieger et al. (2011), Mathieu et al. (2006) and El-Setouhy et al. (2007). Both Brieger et al. (2011) and Newell (1997) investigate treatment for onchocerciasis with ivermectin. Newell (1997) report 4 rounds of treatment in Burundi, while Brieger et al. (2011) investigate the African Programme for Onchocerciasis Control (APOC), studying projects in Nigeria and Cameroon. Mathieu et al. (2006) and El-Setouhy et al. (2007) examine participation in mass drug administration of lymphatic filariasis with DEC and albendazole in Leogane, Haiti and Egypt, respectively.

### 5.2 Data analysis

Only Mathieu et al. (2006) gives the numbers attending all different combinations of rounds (e.g. the percentage of the population attending only rounds 1 and 2, say). From the combinations of rounds in Mathieu et al. (2006) it is straightforward to calculate the coverages of different rounds (round 1 = 60%, round 2 = 62% and round 3 = 68%) and the correlations between different rounds (corr_12_ = 0.5351, corr_13_ = 0.2979 and corr_23_ = 0.5247).

However it is also possible to use the distribution of number of rounds attended, by making the assumption that all rounds are similar. This is a simplifying assumption, that is not generally entirely satisfied, but gives an indication of the required correlations. To use the distribution of number of rounds attended, we define *X*_*i*_ to be a vector of length given by the population size, which is one if that individual attended the drug administration in round *i*, and zero otherwise. Then *Z* = ∑_*i*_*X*_*i*_ gives how many rounds each individual attended. We wish to know the correlations corr(*X*_*i*_, *X*_*j*_) for *i* ≠ *j*. To determine this we use the relationship:

(3) $$\mathrm{var}\left(\sum_{i}X_{i}\right)=\sum_{i}\mathrm{var}(X_{i})+2\sum_{i}\sum_{j=1}^{i}\mathrm{cov}(X_{i},X_{j}).$$

**Fig. 5:**
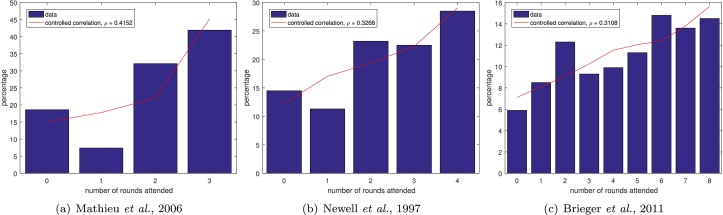
Existing data (blue bars) with controlled correlation scheme distributions using the estimated correlations and coverages (red lines).

Hence if the *X*_*i*_ are identically distributed then var(*X*_*i*_) = var(*X*) for all *i* and $\mathrm{cov}(X_{i_{1}},X_{j_{1}})=\mathrm{cov}(X_{i_{2}},X_{j_{2}})$ for all *i*_1_, *j*_1_, *i*_2_, *j*_2_, and

(4) $$\mathrm{corr}(X_{i},X_{j})=\frac{\mathrm{cov}(X_{i},X_{j})}{\mathrm{var}(X)},$$

(5) $$=\frac{\mathrm{var}(Z)}{M(M+1)\mathrm{var}(X)}-\frac{1}{M+1},$$

where *M* is the number of rounds. We may also calculate var(*X*) from *Z* via the formula

(6) $$E(Z)=E\left(\sum X_{i}\right)=\sum E(X_{i}),$$

hence

(7) $$E(X)=\frac{1}{M}E(Z),$$

and, since *X* is a Bernoulli random variable with mean E(X), then var(X)=E(X)(1−E(X)). For each dataset we calculate the estimated coverage per year and estimated correlation. We plot the data (blue bars in Fig. 5) along with distribution obtained by using these with the controlled correlation scheme (red lines in Fig. 5).

Applying this to the data in Mathieu et al. (2006) we obtain an estimated coverage per year of 66% and an estimated correlation of 0.4152 between years. This seems like a reasonable estimate of both the coverages and the correlations, while clearly not capturing the lower correlation between rounds 1 and 3 seen in the individual-level data. This limitation can also be seen when plotting the distributions (Fig. 5) since the low proportion attending exactly one round is not well captured.

Both Brieger et al. (2011) and Newell (1997) give only the number of rounds attended. Using our technique on the data from Newell (1997) gives an estimated coverage of 60% and a correlation of 0.3268, contrasting with reported coverages of between 40.5% and 49.0% (Newell, 1997). However, the fit obtained by using the estimated coverage and correlation is good, only showing a small overestimate for the percentage attending one round (Fig. 5(b)). Brieger et al. (2011) present a larger number of treatment rounds (Fig. 5(c)), from which we estimate a coverage of 57% and a correlation of 0.3108. This dataset highlights the issue of assuming all rounds are approximately the same, since we would expect coverages to vary over the large number of rounds. Mean coverage rates were only reported for three years: 70% in 2003; 70% in 2004 and 74% in 2005 (Brieger et al., 2011). Given these drawbacks it is perhaps surprising that this dataset seems to show the best fit so far (Fig. 5(c)). This may be due to the larger amount of data that can be fit and the smaller impact of the fluctuations in individual years on the overall fit. In addition, the attendances in this dataset were taken from village registers to avoid reporting bias, which may improve the quality of the dataset, while also indicating that more detailed individual-level data may be available. The discrepancies found by Brieger et al. (2011) between the village registers and the reported coverage levels is indicative of the need to examine the accuracy of coverage reporting and assessment.

**Fig. 6:**
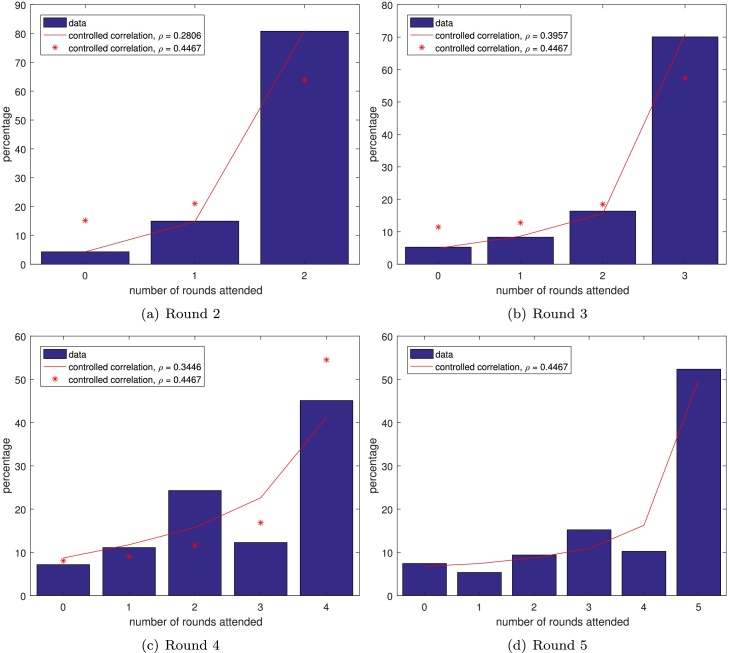
Data (blue bars) from El-Setouhy et al., 2007 with controlled correlation scheme distributions using the estimated correlation up to that round (red lines) and using the estimated correlation from all the rounds (red stars).

Finally, El-Setouhy et al. (2007) reported the number of rounds attended (assessed by a sample survey) after each round of MDA up to a total of 5 (Fig. 6). This gives us the opportunity to calculate our statistical measures over multiple rounds, testing the assumption that the different rounds are roughly the same. The mean coverages found after each year were 82.41%, 88.24%, 83.74%, 69.26% and 74.51%, which were a little lower than those reported (86.7%, 95.5%, 90.1% and 88.8% for rounds 1–4, while coverage was not reported for round 5). Note that the two values are not exactly comparable for each round since, for example, in round 4, the mean coverage is averaged over rounds 1–4, whereas the reported coverage is just for that year. It should also be noted that, since the people surveyed were different after each round, the reported data is in fact inconsistent, with the percentage of people receiving zero rounds of treatment increasing over time. The estimated average correlation between rounds was found (using equation (5)) to be 0.2806, 0.3957, 0.3446 and 0.4467 after rounds 2, 3, 4 and 5, respectively. This would imply that the level of systematic noncompliance increases over time, which is somewhat intuitive: one might expect that after multiple rounds of MDA people get into the habit of attending or not attending.

**Fig. 7:**
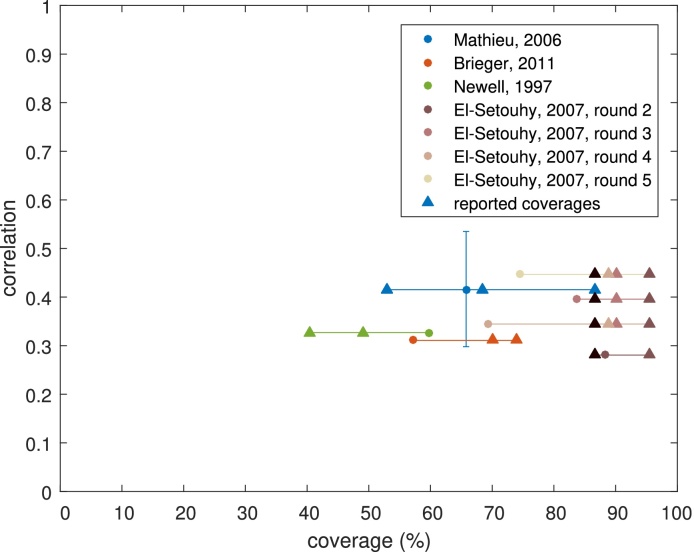
An overview of the datasets obtained with calculated average coverages and correlations (coloured circles) and reported coverages (coloured triangles). For the El-Setouhy et al. (2007) dataset the colours refer to the round that the data is taken from, so that the triangles give the reported coverage for rounds 1–4, while the circles represent the calculated average coverages and correlations after 2–5 rounds. The horizontal lines demonstrate which reported coverages refer to which calculated values, while the vertical line for Mathieu et al. (2006) shows the range of correlations found when using the full dataset (which reports which rounds people attended, rather than just how many rounds).

The range of values taken by the data is shown in Fig. 7 with calculated average coverages and correlations (coloured circles) and reported coverages (coloured triangles). We can see from this that our calculated coverages can be systematically higher or lower than the reported coverages but, with the exception of the El-Setouhy et al. (2007) data, are not a large deviation. In addition, while the coverages in our data range from 40.5% to 95.5%, the range of correlations found is quite narrow (between 0.2806 and 0.5351). Thus there is some evidence that correlations may be approximately the same, even in data from different years, countries, diseases and administered drugs. We show the distribution of number of rounds attended for our controlled correlation model with correlation 0.4 in Fig. 2(h) for comparison with the other schemes.

## 6 Discussion

Systematic non-adherence is clearly an important factor in the success or failure of an MDA campaign, but it's impact depends on the extent of the permanent or occasional lack of treatment for these groups, and the size of those groups. Therefore, the conclusions of modelling studies depend importantly on the underlying assumptions about this behaviour. Here we have summarised the different ways of modelling systematic non-adherence, showing the range of different assumptions that have been made in the modelling literature. Individual-based modellers were the first to introduce systematic non-adherence, making use of their model's flexibility in characterising individual behaviours (Plaisier et al., 1990, Plaisier et al., 1998, de Vlas et al., 1996). More recently, compartmental, deterministic models have been adapted to use a variety of methodologies for representing this behaviour, each of which have particular limitations (Basá nez and Boussinesq, 1999, Turner et al., 2013). Here we have introduced a new, more flexible way of including this effect in mathematical models. Our proposed variable correlation scheme allows the explicit inclusion of a correlation between rounds, but the scheme as proposed requires the coverage levels to remain the same over multiple rounds and the correlations between any two rounds to be the same. We note that the scheme may easily be extended using techniques by Qaqish (2003) to produce specified coverage levels and/or a specified correlation matrix between rounds.

Using simplified models of infection, we investigated the impact of different assumptions on infection rates, coverage and systematic non-adherence and conclude that the effect of systematic non-adherence is more extreme at lower rates of infection. It appears that the effects are slightly lower in helminth models compared to the SIS model, however this may be due to the parameter values chosen. We note that more complicated models of helminth dynamics, in which different assumptions are taken for each specific helminth species may affect this result. More work is needed to fully understand how the impact of treatments on the probability of disease transmission may change the effects of systematic non-adherence.

In the case where non-adherence to treatment is correlated with infection risk, such as in sub-populations with poor sanitation and poor access to health-care, then this generally leads to higher prevalences in the long run. However in this situation, surprisingly, it is better to focus on treating people who are not at risk of infection early in the program, since they are more likely to remain uninfected after being cured.

Although there are only a few studies characterising the extent of systematic non-adherence, we have demonstrated that gathering data about the number of rounds that people attend can be used to determine the correlation between years, which is needed to parameterise models of systematic non-adherence. Despite our (small amount of) data coming from different sources, types of infections, years and countries, the parameter values we obtain are relatively similar (with correlations between 0.28 and 0.54). More data of this type needs to be collected to dramatically increase the accuracy of our predictions. While there are concerns about the quality of data achievable (particularly the ability of individuals to accurately report the number of rounds of treatment they have received, Brieger et al., 2011, Shuford et al., 2016), even small additional information could be potentially useful and the collection of such data should be informed by the local context. Whilst we focus on a general description, it is important to identify and quantify the social and logistical drivers in order to overcome them. It is important to note that correlations in different geographical areas may be a way of prediction where hotspots are most likely to occur.

## 7 Conclusions

Overall this study highlights the importance of careful consideration of the drivers and characteristics of systematic non-adherence, and of model comparison, so that different predictions can be evaluated in terms of their parameter and structural assumptions. Further work should focus in two main areas: gathering data and extending analytical tools to quantify the extent of systematic non-adherence; and expanding current and future models to include and analyse these effects. We do not make claims for any particular diseases in this work, but instead demonstrate that systematic non-adherence can have a large effect and encourage others to investigate these effects in their own disease- and country-specific circumstances.

## References

[bib0005] Anderson R.M., Hollingsworth T.D., Truscott J., Brooker S.J. (2012). Optimisation of mass chemotherapy to control soil-transmitted helminth infection. Lancet.

[bib0010] Anderson R.M., Turner H.C., Farrell S.H., Yang J., Truscott J.E. (2015). What is required in terms of mass drug administration to interrupt the transmission of schistosome parasites in regions of endemic infection?. Parasit. Vectors.

[bib0015] Basá nez M.G., Boussinesq M. (1999). Population biology of human onchocerciasis. Philos. Trans. R. Soc. Lond. Ser. B Biol. Sci..

[bib0020] Blok D.J., de Vlas S.J., Richardus J.H. (2015). Global elimination of leprosy by 2020: are we on track. Parasit. Vectors.

[bib0025] Boyd A., Won K.Y., McClintock S.K., Donovan C.V., Laney S.J., Williams S.A., Pilotte N., Streit T.G., Beau De Rochars M.V.E., Lammie P.J. (2010). A community-based study of factors associated with continuing transmission of lymphatic filariasis in Leogane, Haiti. PLoS Neglect. Trop. Dis..

[bib0030] Brieger W.R., Okeibunor J.C., Abiose A.O., Ndyomugyenyi R., Wanji S., Elhassan E., Amazigo U.V. (2012). Characteristics of persons who complied with and failed to comply with annual ivermectin treatment. Trop. Med. Int. Health.

[bib0035] Brieger W.R., Okeibunor J.C., Abiose A.O., Wanji S., Elhassan E., Ndyomugyenyi R., Amazigo U.V. (2011). Compliance with eight years of annual ivermectin treatment of onchocerciasis in Cameroon and Nigeria. Parasit. Vectors.

[bib0040] Brooker S.J., Kabatereine N.B., Fleming F., Devlin N. (2008). Cost and cost-effectiveness of nationwide school-based helminth control in Uganda: intra-country variation and effects of scaling-up. Health Policy Plan..

[bib0045] Coffeng L.E., Bakker R., Montresor A., de Vlas S.J. (2015). Feasibility of controlling hookworm infection through preventive chemotherapy: a simulation study using the individual-based WORMSIM modelling framework. Parasit. Vectors.

[bib0050] Coffeng L.E., Stolk W.A., Hoerauf A., Habbema D., Bakker R., Hopkins A.D., de Vlas S.J. (2014). Elimination of African onchocerciasis: Modeling the impact of increasing the frequency of ivermectin mass treatment. PLoS ONE.

[bib0055] Cromwell E.A., Ngondi J., Gatpan G., Becknell S., Kur L., McFarland D., King J.D., Emerson P.M. (2009). Estimation of population coverage for antibiotic distribution for trachoma control: a comparison of methods. Int. Health.

[bib0060] de Vlas S.J., van Oortmarssen G.J., Gryseels B., Polderman A.M., Plaisier A.P., Habbema J.D. (1996). SCHISTOSIM: a microsimulation model for the epidemiology and control of schistosomiasis. Am. J. Trop. Med. Hygiene.

[bib0065] El-Setouhy M., Elaziz K.M.A., Hanan H., Farid H.A., Kamal H.A., Ramzy R.M.R., Shannon W.D., Weil G.J. (2007). The effect of compliance on the impact of mass drug administration for elimination of lymphatic filariasis in Egypt. Am. J. Trop. Med. Hygiene.

[bib0070] Farrell S.H., Truscott J.E., Anderson R.M. (2017). The importance of patient compliance in repeated rounds of mass drug administration (MDA) for the elimination of intestinal helminth transmission. Parasit. Vectors.

[bib0075] Gambhir M., Pinsent A. (2015). Possible changes in the transmissibility of trachoma following MDA and transmission reduction: implications for the GET2020 goals. Parasit. Vectors.

[bib0080] Gillespie D.T. (1977). Exact stochastic simulation of coupled chemical reactions. J. Phys. Chem..

[bib0085] Griffin J.T., Hollingsworth T.D., Okell L.C., Churcher T.S., White M., Hinsley W., Bousema T., Drakeley C.J., Ferguson N.M., Basáñez M.G., Ghani A.C. (2010). Reducing Plasmodium falciparum malaria transmission in Africa: a model-based evaluation of intervention strategies. PLoS Med..

[bib0090] Gurarie D., Yoon N., Li E., Ndeffo-Mbah M., Durham D., Phillips A.E., Aurelio H.O., Ferro J., Galvani A.P., King C.H. (2015). Modelling control of *Schistosoma haematobium* infection: predictions of the long-term impact of mass drug administration in Africa. Parasit. Vectors.

[bib0095] Holland C.V., O’Shea E., Asaolu S.O., Turley O., Crompton D.W. (1996). A cost-effectiveness analysis of anthelminthic intervention for community control of soil-transmitted helminth infection: levamisole and *Ascaris lumbricoides*. J. Parasitol..

[bib0100] Irvine M.A., Reimer L.J., Njenga S.M., Gunawardena S., Kelly-Hope L., Bockarie M., Hollingsworth T.D. (2015). Modelling strategies to break transmission of lymphatic filariasis-aggregation, adherence and vector competence greatly alter elimination. Parasit. Vectors.

[bib0105] Jambulingam P., Subramanian S., de Vlas S.J., Vinubala C., Stolk W.A. (2016). Mathematical modelling of lymphatic filariasis elimination programs in India: required duration of mass drug administration and post-treatment level of infection indicators. Parasit. Vectors.

[bib0110] King J.D., Zielinski-Gutierrez E., Pa’au M., Lammie P. (2011). Improving community participation to eliminate lymphatic filariasis in American Samoa. Acta Trop..

[bib0115] Krentel A., Fischer P.U., Weil G.J. (2013). A review of factors that influence individual compliance with mass drug administration for elimination of lymphatic filariasis. PLoS Neglect. Trop. Dis..

[bib0120] Krentel A., Damayanti R., Titaley C.R., Suharno N., Bradley M., Lynam T. (2016). Improving coverage and compliance in mass drug administration for the elimination of LF in two ‘endgame’ districts in Indonesia using micronarrative surveys. PLoS Neglect. Trop. Dis..

[bib0125] Liu F., Porco T.C., Amza A., Kadri B., Nassirou B., West S.K., Bailey R.L., Keenan J.D., Lietman T.M. (2015). Short-term forecasting of the prevalence of clinical trachoma: utility of including delayed recovery and tests for infection. Parasit. Vectors.

[bib0130] Mathieu E., Direny A.N., de Rochars M.B., Streit T.G., Addiss D.G., Lammie P.J. (2006). Participation in three consecutive mass drug administrations in Leogane, Haiti. Trop. Med. Int. Health.

[bib0135] Mpanya A., Hendrickx D., Vuna M., Kanyinda A., Lumbala C., Tshilombo V., Mitashi P., Luboya O., Kande V., Boelaert M., Lefèvre P., Lutumba P. (2012). Should I get screened for sleeping sickness? A qualitative study in Kasai province, Democratic Republic of Congo. PLoS Neglect. Trop. Dis..

[bib0140] Newell E.D. (1997). Effect of mass treatments with ivermectin, with only partial compliance, on prevalence and intensity of *O. volvulus* infection in adults and in untreated 4 and 5 year-old children in Burundi. Trop. Med. Int. Health.

[bib0145] Okell L.C., Griffin J.T., Kleinschmidt I., Hollingsworth T.D., Churcher T.S., White M.J., Bousema T., Drakeley C.J., Ghani A.C. (2011). The potential contribution of mass treatment to the control of plasmodium falciparum malaria. PLoS ONE.

[bib0150] Pandey A., Atkins K.E., Bucheton B., Camara M., Aksoy S., Galvani A.P., Ndeffo-Mbah M.L. (2015). Evaluating long-term effectiveness of sleeping sickness control measures in Guinea. Parasit. Vectors.

[bib0155] Parker M., Allen T. (2013). Does mass drug administration for the integrated treatment of neglected tropical diseases really work? Assessing evidence for the control of schistosomiasis and soil-transmitted helminths in Uganda. Health Res. Policy Syst..

[bib0160] Parker M., Allen T. (2013). Will mass drug administration eliminate lymphatic filariasis? Evidence from northern coastal Tanzania. J. Biosoc. Sci..

[bib0165] Plaisier A.P., Das P.K., Souza W., Lapa T., Furtado A.F., van der Ploeg C.P.B., Habbema J.D.F., van Oortmarssen G.J. (1998). The LYMFASIM simulation program for modelling lymphatic filariasis and its control. Methods Inf. Med..

[bib0170] Plaisier A.P., Stolk W.A., van Oortmarssen G.J., Habbema J.D.F. (2000). Effectiveness of annual ivermectin treatment for *Wuchereria bancrofti* infection. Parasitol. Today.

[bib0175] Plaisier A.P., van Oortmarssen G.J., Habbema J.D.F., Remme J., Alley E.S. (1990). ONCHOSIM: a model and computer simulation program for the transmission and control of onchocerciasis. Comput. Methods Prog. Biomed..

[bib0180] Qaqish B.F. (2003). A family of multivariate binary distributions for simulating correlated binary variables with specified marginal means and correlations. Qual. Res..

[bib0185] Rock K.S., Torr S.J., Lumbala C., Keeling M.J. (2015). Quantitative evaluation of the strategy to eliminate human African trypanosomiasis in the Democratic Republic of Congo. Parasit. Vectors.

[bib0190] Roy R.N., Sarkar A.P., Misra R., Chakroborty A., Mondal T.K., Bag K. (2013). Coverage and awareness of and compliance with mass drug administration for elimination of lymphatic filariasis in Burdwan District, West Bengal, India. J. Health Popul. Nutr..

[bib0195] Shuford K.V., Turner H.C., Anderson R.M. (2016). Compliance with anthelmintic treatment in the neglected tropical diseases control programmes: a systematic review. Parasit. Vectors.

[bib0200] Singh B.K., Michael E. (2015). Bayesian calibration of simulation models for supporting management of the elimination of the macroparasitic disease, lymphatic filariasis. Parasit. Vectors.

[bib0205] Slater H.C., Walker P.G.T., Bousema T., Okell L.C., Ghani A.C. (2014). The potential impact of adding ivermectin to a mass treatment intervention to reduce malaria transmission: a modelling study. J. Infect. Dis..

[bib0210] Stolk W.A., de Vlas S.J., Borsboom G.J.J.M., Habbema J.D.F. (2008). LYMFASIM, a simulation model for predicting the impact of lymphatic filariasis control: quantification for African villages. Parasitology.

[bib0215] Stolk W.A., Subramanian S., van Oortmarssen G.J., Das P.K., Habbema J.D.F. (2003). Prospects for elimination of bancroftian filariasis by mass drug treatment in Pondicherry, India: a simulation study. J. Infect. Dis..

[bib0220] Stolk W.A., Walker M., Coffeng L.E., Basá nez M.G., de Vlas S.J. (2015). Required duration of mass ivermectin treatment for onchocerciasis elimination in Africa: a comparative modelling analysis. Parasit. Vectors.

[bib0225] Subramanian S., Stolk W.A., Ramaiah K.D., Plaisier A.P., Krishnamoorthy K., van Oortmarssen G.J., Amalraj D.D., Habbema J.D.F., Das P.K. (2004). The dynamics of *Wuchereria bancrofti* infection: a model-based analysis of longitudinal data from Pondicherry, India. Parasitology.

[bib0230] Tekle A.H., Zouré H.G.M., Noma M., Boussinesq M., Coffeng L.E., Stolk W.A., Remme J.H.F. (2016). Progress towards onchocerciasis elimination in the participating countries of the African Programme for Onchocerciasis Control: epidemiological evaluation results. Infect. Dis. Poverty.

[bib0235] Truscott J.E., Turner H.C., Anderson R.M. (2015). What impact will the achievement of the current World Health Organisation targets for anthelmintic treatment coverage in children have on the intensity of soil transmitted helminth infections?. Parasit. Vectors.

[bib0240] Turner H.C., Churcher T.S., Walker M., Osei-atweneboana M.Y., Prichard R.K. (2013). Uncertainty surrounding projections of the long-term impact of ivermectin treatment on human onchocerciasis. PLoS Neglect. Trop. Dis..

[bib0245] Turner H.C., Walker M., Attah S.K., Opoku N.O., Awadzi K., Kuesel A.C., Basáñez M.G. (2015). The potential impact of moxidectin on onchocerciasis elimination in Africa: an economic evaluation based on the Phase II clinical trial data. Parasit. Vectors.

[bib0250] Turner H.C., Walker M., Churcher T.S., Basá nez M.G. (2014). Modelling the impact of ivermectin on River Blindness and its burden of morbidity and mortality in African Savannah: EpiOncho projections. Parasit. Vectors.

[bib0255] Turner H.C., Walker M., Churcher T.S., Osei-Atweneboana M.Y., Biritwum N.K., Hopkins A.D., Prichard R.K., Basá nez M.G. (2014). Reaching the London declaration on neglected tropical diseases goals for onchocerciasis: an economic evaluation of increasing the frequency of ivermectin treatment in Africa. Clin. Infect. Dis..

[bib0260] Winnen M., Plaisier A.P., Alley E.S., Nagelkerke N.J.D., van Oortmarssen G., Boatin B.A., Habbema J.D.F. (2002). Can ivermectin mass treatments eliminate onchocerciasis in Africa?. Bull. World Health Organ..

[bib0265] World Health Organization (2013). Sustaining the Drive to Overcome the Global Impact of Neglected Tropical Diseases. Second WHO Report on Neglected Tropical Diseases.

[bib0270] World Health Organization (2015). Global Programme to Eliminate Lymphatic Filariasis: Progress Report, 2014 Weekly Epidemiological Record.

