# Supplementary material for: Measuring and modelling the effects of systematic non-adherence to mass drug administration
Source: Epidemics. 2017 Mar;18:56–66. doi: 10.1016/j.epidem.2017.02.002 (PMC5340860; doi:10.1016/j.epidem.2017.02.002)
Supplement: Supplementary file 1 [file mmc1.pdf]

Systematic non-adherence: supplementary material

1 Correlations in previously-used models

1.1 Random model

In the random model in round i we have:

$$X_i = \begin{cases} 1, & \text{with probability } c, \\ 0, & \text{else,} \end{cases} \tag{1}$$

So the mean and variance are given by

$$\begin{aligned} \mathbb{E}(X_i) &= c, &(2)\\ \mathrm{var}(X_i) &= \mathbb{E}(X_i^2) - \mathbb{E}(X_i)^2, &(3)\\ &= c(1-c), &(4) \end{aligned}$$

and the covariance between rounds is given by

$$\begin{aligned} \mathrm{cov}(X_i, X_j) &= \mathbb{E}(X_i X_j) - \mathbb{E}(X_i)\mathbb{E}(X_j), &(5)\\ &= c^2 - c^2, &(6)\\ &= 0, &(7) \end{aligned}$$

so that the correlation is also 0.

1.2 Systematic model

In the random model in round i we have a subpopulation of size $(1-c)$ that never attend treatment and a subpopulation of size c that always attend. Since each individual does the same thing in each round, the correlation between rounds is exactly 1.

1.3 Semi-systematic model

In the 'semi-systematic' model in round i we have:

$$X_i = \begin{cases} 1, & \text{with probability } a^{\frac{1-c}{c}}, \\ 0, & \text{else,} \end{cases} \tag{8}$$

where a is a uniformly distributed random number on $[0, 1]$. Now the mean is given by

$$\begin{aligned} \mathbb{E}(X_i) &= \int_0^1 a^{\frac{1-c}{c}} \, \mathrm{d}\, a, &(9)\\ &= c, &(10) \end{aligned}$$

and the variance by

$$\begin{aligned} \mathrm{var}(X_i) &= \mathbb{E}(X_i^2) - \mathbb{E}(X_i)^2, &(11)\\ &= \int_0^1 a^{\frac{1-c}{c}} \, \mathrm{d}\, a - c^2, &(12)\\ &= c(1-c), &(13) \end{aligned}$$

and the covariance between rounds is given by

$$\begin{aligned}
\mathrm{cov}(X_i, X_j) &= \mathbb{E}(X_i X_j) - \mathbb{E}(X_i)\mathbb{E}(X_j), && (14)\\
&= \int_0^1 a^{\frac{1-c}{c}} a^{\frac{1-c}{c}} \,\mathrm{d}\, a - c^2, && (15)\\
&= \frac{c}{c-2} - c^2, && (16)
\end{aligned}$$

so that the correlation is

$$\begin{aligned}
\mathrm{corr}(X_i, X_j) &= \frac{\mathrm{cov}\, X_i, X_j}{\sqrt{\mathrm{var}\, X_i \,\mathrm{var}\, X_j}}, && (17)\\
&= \frac{\frac{c}{c-2} - c^2}{c(1-c)}, && (18)\\
&= \frac{1-c}{2-c}. && (19)
\end{aligned}$$

1.4 Griffin, 2010 variable correlation schemes

This scheme was first introduced by Griffin et al. (2010) and subsequently used by Irvine et al. (2015). The authors used a method where increasing a parameter, ρ increased the correlation between rounds, by giving each individual, i, a normally distributed random number $u_i \sim N(u_0, \sigma^2)$. Then in each round individual i is treated if $z \sim N(u_i, 1)$ is less than zero. So if u_i is very large and positive (or negative) then individual i is very likely to draw positive (respectively, negative) $z \sim N(u_i, 1)$. So the width of the distribution $N(u_0, \sigma^2)$ controls the 'systematicness' of the treatment campaign. To have a given coverage, c, requires $u_0 = -\Phi^{-1}(c)\sqrt{1+\sigma^2}$, and for a correlation of ρ between the values of z in different rounds we require $\sigma^2 = \rho/(1-\rho)$. So for $\rho = 0$ the z are uncorrelated between rounds, whereas for $\rho = 1$ the z are completely correlated between rounds. However, the random variable given by whether z is less than zero does not have correlation ρ.

1.5 Controlled correlation scheme

1.5.1 Method

We wish to generate a sequence of random variables $\mathbf{Y_i} = (y_i^1, \ldots, y_i^N)$, (where N is the population size and $y_i = \{0, 1\}$) in which $\mathbb{E}(\mathbf{Y_i}) = \mu_y \mathbb{I}$, $\forall i$ and $corr(y_i^k, y_j^k) = \rho$, $\forall i, j, k$. We do this using the following algorithm: for round 1, take $Y_1 = \text{Bernouilli}(\mu_y)$; then for subsequent rounds take $Y_i = \text{Bernouilli}(\lambda_i)$, where

$$\lambda_i = \frac{\mu_y(1-\rho) + \rho \mathbf{R_i}}{1 + (i-2)\rho}, \qquad (20)$$

and $\mathbf{R_i} = \sum_{j=1}^{i-1} \mathbf{Y_i}$, (i.e. the kth element is the number of rounds attended so far by individual k after $i-1$ rounds).

1.5.2 Proof

Let $\mathbf{X_i} = (\mathbf{Y_1}, \ldots, \mathbf{Y_{i-1}})^T$, and $\mathbb{E}(\mathbf{Y_i}|\mathbf{X_i} = \mathbf{x_i}) = \mu_y + b_i^T(x_i - \mathbb{E}(\mathbf{X_i}))$. Then

$$\begin{aligned}
cov(\mathbf{X_i}, \mathbf{Y_i}) &= \mathbb{E}\left[(\mathbf{X_i} - \mathbb{E}(\mathbf{X_i}))(\mathbf{Y_i} - \mu_y)\right], && (21)\\
&= \sum_{\mathbf{x_i}} (\mathbf{x_i} - \mathbb{E}(\mathbf{X_i}))(1-\mu_y) P(\mathbf{X_i} = \mathbf{x_i})(\mu_y + b_i^T(\mathbf{x_i} - \mathbb{E}(\mathbf{X_i})))\\
&\quad \sum_{\mathbf{x_i}} -(\mathbf{x_i} - \mathbb{E}(\mathbf{X_i}))\mu_y P(\mathbf{X_i} = \mathbf{x_i})\left[1 - (\mu_y + b_i^T(\mathbf{x_i} - \mathbb{E}(\mathbf{X_i})))\right], && (22)\\
&= \sum_{\mathbf{x_i}} (\mathbf{x_i} - \mathbb{E}(\mathbf{X_i})) P(\mathbf{X_i} = \mathbf{x_i}) b_i^T(\mathbf{x_i} - \mathbb{E}(\mathbf{X_i})), && (23)\\
&= cov\left(\mathbf{X_i}, b_i^T \mathbf{X_i}\right), && (24)\\
&= b_i^T cov(\mathbf{X_i}, \mathbf{X_i}). && (25)
\end{aligned}$$

So to achieve some given covariance matrix $s_i := cov(\mathbf{X_i}, \mathbf{Y_i})$ we need to solve $b_i^T = s_i G_i^{-1}$, where $G_i = cov(\mathbf{X_i}, \mathbf{X_i})$.

In our case, since we want a correlation of ρ then the required covariance is given by $s_i = cov(\mathbf{X_i}, \mathbf{Y_i}) = (cov(\mathbf{Y_i}, \mathbf{Y_1}), \ldots, cov(\mathbf{Y_i}, \mathbf{Y_{i-1}})) = (\rho\sigma, \ldots, \rho\sigma)^T$, where $\sigma^2 = \mu_y(1-\mu_y)$ is the variance of Y_i. The matrix G_i is defined by

$$(G_i)_{jk} = cov(\mathbf{Y_j}, \mathbf{Y_k}) = \begin{cases} \rho\sigma, & \text{for } j \neq k, \\ \sigma, & \text{for } j = k. \end{cases} \tag{26}$$

The inverse is given (Poularikas, 1998) by

$$G_i^{-1} = \frac{1}{\sigma(1-\rho)} \left(\mathbb{I} - \frac{\rho \mathbb{J}}{1 + (i-2)\rho} \right), \tag{27}$$

where $\mathbb{I}$ is the identity matrix and $\mathbb{J}$ is a matrix of ones. Hence

$$(b_i)_j = G_i^{-1} s_i = \frac{\rho}{1 + (i-2)\rho}, \ \forall j, \tag{28}$$

and the conditional expectation (or, equivalently for a Bernouilli random variable, the probability of success) is given by

$$\mu_y + b_i^T (x_i - \mathbb{E}(\mathbf{X_i})) = \frac{\mu_y(1-\rho) + \rho \sum_{j=1}^{i-1} y_j}{1 + (i-2)\rho}. \tag{29}$$

In addition, $\mathbb{E}(\mathbf{Y_1}) = \mu_y$, as required, and if $\mathbb{E}(\mathbf{Y_j}) = \mu_y$ for $j = 1, ldots, i-1$ then

$$\begin{aligned} \mathbb{E}(\mathbf{Y_i}) &= \mu_y + \sum_{j=1}^{i-1} \sum_y b_{ij}(y - \mu_j) P(\mathbf{Y_j} = y), && (30) \\ &= \mu_y + \sum_{j=1}^{i-1} b_i^T \mathbb{E}((\mathbf{Y_j} - \mu_y)), && (31) \\ &= \mu_y, && (32) \end{aligned}$$

and so by induction $\mathbb{E}((\mathbf{Y_i}) = \mu_y \ \forall i$, as required.

1.5.3 Equivalence to a simpler scheme

In fact it turns out that this scheme is equivalent to giving each person a parameter that gives their probability of attending any round (which is fixed for that person), but drawing that parameter from a Beta distribution with parameters $\alpha = \mu_y(1-\rho)/\rho$ and $\beta = (1-\mu_y)(1-\rho)/\rho$. This can be seen by de Finetti's theorem, since the scheme produces an exchangeable sequence of random variables (i.e. ones where the joint distribution between any two rounds is the same). De Finetti's theorem states that any exchangeable sequence of random variables can be rewritten by giving each individual a parameter that gives their probability of attending any round. Since our scheme changes probabilities over different rounds in a manner equivalent to a Polya urn model, our probabilities must be drawn from a Beta distribution, and the parameters can be found by equating the mean and correlations from the two methods.

1.5.4 Correlation with other random variables

If $\mathbf{Y_i}$ is a Bernouilli random variable with parameter λ_i, where

$$\lambda_i = \frac{\mu_y(1-\rho) + \rho \mathbf{R_i}}{1 + (i-2)\rho}, \tag{33}$$

and we take a random variable $\mathbf{Z_i}$ with mean (conditioned on the value of $\mathbf{Y_i}$) of η_i and variance σ_z where

$$\eta_i = \mu_z + c(y_i - \mu_y), \tag{34}$$

where μ_z is the desired mean of $\mathbf{Z_i}$. Now

$$\begin{aligned} cov(\mathbf{Z_i}, \mathbf{Y_i}) &= \lambda_i(1-\mu_y)\mathbb{E}(\mathbf{Z_i}-\mu_z|\mathbf{Y_i}=1) - (1-\lambda_i)\mu_y\mathbb{E}(\mathbf{Z_i}-\mu_z|\mathbf{Y_i}=1), &(35)\\ &= \lambda_i(1-\mu_y)c(1-\mu_y) - (1-\lambda_i)\mu_y\mu_y c, &(36)\\ &= c(\lambda_i - 2\lambda_i\mu_y + \mu_y^2). &(37)\end{aligned}$$

So if we want $corr(\mathbf{Z_i}, \mathbf{Y_i}) = \rho_z$ then we take

$$c = \frac{\rho_z\sigma_z\sqrt{\mu_y(1-\mu_y)}}{\lambda_i - 2\lambda_i\mu_y + \mu_y^2)}. \tag{38}$$

since $corr(\mathbf{Z_i}, \mathbf{Y_i}) = cov(\mathbf{Z_i}, \mathbf{Y_i})/(var(\mathbf{Z_i})var(\mathbf{Y_i}))$.

Hence for a binary random variable then, we take $\mathbf{Z_i}$ to be a Bernouilli random variable with parameter η_i, where

$$\eta_i = \mu_z + \frac{\rho_z\sqrt{\mu_z(1-\mu_z)}\sqrt{\mu_y(1-\mu_y)}}{\lambda_i - 2\lambda_i\mu_y + \mu_y^2)}(y_i - \mu_y). \tag{39}$$

Whereas for a gamma distributed random variable with mean μ_z and variance σ_z then

$$\eta_i = \mu_z + \frac{\rho_z\sigma_z\sqrt{\mu_y(1-\mu_y)}}{\lambda_i - 2\lambda_i\mu_y + \mu_y^2)}(y_i - \mu_y). \tag{40}$$

and $\mathbf{Z_i}$ has scale parameter

$$\theta_i = \frac{\sigma_z}{\eta_i}, \tag{41}$$

and shape parameter

$$k_i = \frac{\eta_i}{\theta_i}. \tag{42}$$

1.6 Correlations between treatment and infection risk

We approach this using a very simple model, in which each individual i has some probability T_i of receiving treatment, and acquires disease at some rate β_i, then their probability $P_i(t)$ of being infected at time t is given by

$$\begin{aligned} \frac{dP_i}{dt} &= \beta_i(1-P_i) - T_iP_i, &(43)\\ P_i(T_i,\beta_i,t) &= \frac{\beta_i + T_ie^{-t(T_i+\beta_i)}}{\beta_i + T_i}. &(44)\end{aligned}$$

To find the prevalence in the population we want to find the mean value of P_i over $f(T_i,\beta_i)$, the joint distribution of T_i and β_i. If the $f(T_i,\beta_i)$ is sufficiently tightly distributed around its mean values, then we may take a Taylor expansion around those values:

$$\begin{aligned}\langle P_i(T_i,\beta_i,t)\rangle \approx&\langle P_i(\bar{T},\bar{\beta}) + (T_i-\bar{T})\frac{\partial P_i}{\partial T}(\bar{T},\bar{\beta}) + (\beta_i-\bar{\beta})\frac{\partial P_i}{\partial \beta}(\bar{T},\bar{\beta})\\ &+\frac{1}{2}(T_i-\bar{T})^2\frac{\partial^2 P_i}{\partial T^2}(\bar{T},\bar{\beta}) + (T_i-\bar{T})(\beta_i-\bar{\beta})\frac{\partial^2 P_i}{\partial T\partial\beta}(\bar{T},\bar{\beta}) + \frac{1}{2}(\beta_i-\bar{\beta})^2\frac{\partial^2 P_i}{\partial \beta^2}(\bar{T},\bar{\beta})\rangle,\\ =&P_i(\bar{T},\bar{\beta}) + \frac{1}{2}\frac{\partial^2 P_i}{\partial T^2}(\bar{T},\bar{\beta})var(T) + \frac{\partial^2 P_i}{\partial T\partial\beta}(\bar{T},\bar{\beta})corr(T,\beta)\sqrt{var(T)}\sqrt{var(\beta)} + \frac{1}{2}\frac{\partial^2 P_i}{\partial \beta^2}(\bar{T},\bar{\beta})var(\beta),\end{aligned} \tag{45}$$

where $\bar{\beta}$ and $\bar{T}$ are the mean values of β and T, respectively. This allows us to assess the impact of the correlation between T and β on the overall prevalence. In particular,

$$\frac{\partial^2 P_i}{\partial T\partial\beta}(\bar{T},\bar{\beta}) = \frac{e^{-t(\beta+T)}}{(\beta+T)^3}\left(-\beta + t^2T^3 + 2\beta t^2T^2 + \beta^2t^2T - \beta^2t + tT^2 + (\beta-T)e^{t(\beta+T)} + T\right), \tag{46}$$

and as $t \to \infty$

$$\frac{\partial^2 P_i}{\partial T \partial \beta}(\bar{T}, \bar{\beta}) \to \frac{\beta - T}{(\bar{\beta} + \bar{T})^3}, \quad (47)$$

so positive correlation decreases prevalence if, and only if, treatment is more common than infection (i.e. if $\bar{T} > \bar{\beta}$). This is intuitively understandable, since if the population is being treated faster than becoming infected then it is better to treat the subpopulation that is becoming infected faster. Conversely if the population is becoming reinfected faster than it is possible to treat them, then it is more effective to focus on the population that are reinfected slowly (i.e. having negative correlation between treatment and infection).

Interestingly, equation (46) is always positive for small values of t, implying that positive correlation between treatment and infection always initially increases prevalence. This can be understood, since at the beginning of a treatment campaign it is better to focus on the 'easy gains' by treating those people that will not quickly become reinfected. The time at which one should switch from focussing on low-risk to high-risk individuals can be approximately calculated, for any particular parameter set, from equation (46).

Since this is a very simplified system, which does not include even basic effects such as transmission from infected individuals to susceptibles, it is unclear whether these results will hold in more realistic models. To investigate this we plot simulation timecourses and elimination times for our SIS and helminth simulation models (Figure 1). It is clear that the elimination times do increase with negative correlation between infection risk and the probability of treatment (Figure 1(b) and (d)). In addition we can see from the timecourses, that at early times this ordering is reversed, and only becomes clear after some time (Figure 1(a) and (c)). These simulations therefore are in agreement with our analytical predictions. We note that, while the long term effects of correlations are enhanced at lower reinfection rates (β), the reverse ordering after the first round of treatment is enhanced during the first round of reinfection, and is greater for higher reinfection rates.

We may also calculate how the variance of β and T affects the steady state prevalence. Differentiating equation (45) with respect to $var(\beta)$, we obtain

$$\frac{1}{2}\frac{\partial^2 P_i}{\partial T^2}(\bar{T}, \bar{\beta}) + \frac{1}{2\sqrt{var(\beta)}} corr(T, \beta)\sqrt{var(T)} \frac{\partial^2 P_i}{\partial T \partial \beta}(\bar{T}, \bar{\beta}) \to \frac{corr(\beta, T)\sqrt{var(T)}(\bar{\beta} - \bar{T}) - 2\bar{T}\sqrt{var(\beta)}}{2\sqrt{var(\beta)}(\bar{\beta} + \bar{T})^3}, \quad (48)$$

as $t \to \infty$. So if $\bar{T} > \bar{\beta}$, with high-risk individuals more likely to be treated (i.e. $corr(\beta, T) > 0$), then an increase in the variance of infection decreases the prevalence. If $\bar{T} < \bar{\beta}$, but with high-risk individuals less likely to be treated (i.e. $corr(\beta, T) < 0$), then an increase in the variance of infection rates also decreases the prevalence.

Differentiating equation (45) with respect to $var(T)$ gives

$$\frac{1}{2}\frac{\partial^2 P_i}{\partial T^2}(\bar{T}, \bar{T}) + \frac{1}{2\sqrt{var(T)}} corr(T, \beta)\sqrt{var(\beta)} \frac{\partial^2 P_i}{\partial T \partial \beta}(\bar{T}, \bar{\beta}) \to \frac{corr(\beta, T)\sqrt{var(\beta)}(\bar{\beta} - \bar{T}) + 2\bar{\beta}\sqrt{var(T)}}{2\sqrt{var(T)}(\bar{\beta} + \bar{T})^3}, \quad (49)$$

as $t \to \infty$. So if $\bar{T} < \bar{\beta}$, with high-risk individuals more likely to be treated (i.e. $corr(\beta, T) > 0$), then an increase in the variance of treatment increases the prevalence. If $\bar{T} > \bar{\beta}$, but with high-risk individuals less likely to be treated (i.e. $corr(\beta, T) < 0$), then an increase in the variance of treatment rates also increases the prevalence. Hence if the correlations are as desired from the earlier analysis of the correlation, then it is better to have as low a variance in treatment rates across the population as possible.

References

Griffin, J. T., Hollingsworth, T. D., Okell, L. C., Churcher, T. S., White, M., Hinsley, W., Bousema, T., Drakeley, C. J., Ferguson, N. M., Basáñez, M. G., Ghani, A. C., 2010. Reducing Plasmodium falciparum malaria transmission in Africa: A model-based evaluation of intervention strategies. PLoS Medicine 7 (8).

Irvine, M. A., Reimer, L. J., Njenga, S. M., Gunawardena, S., Kelly-Hope, L., Bockarie, M., Hollingsworth, T. D., 2015. Modelling strategies to break transmission of lymphatic filariasis - aggregation, adherence and vector competence greatly alter elimination. Parasites and Vectors 8, 547. URL http://dx.doi.org/10.1186/s13071-015-1152-3

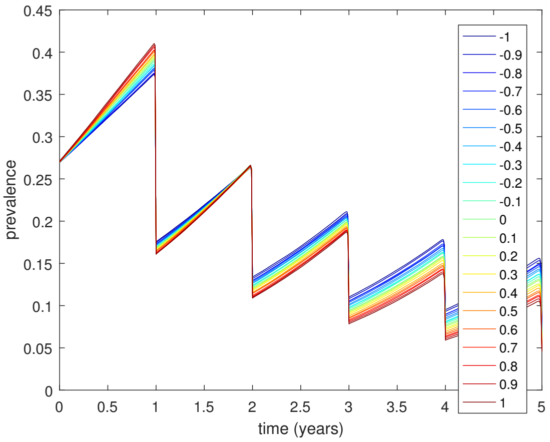

(a) SIS simulation timecourses, $\beta = 0.8$

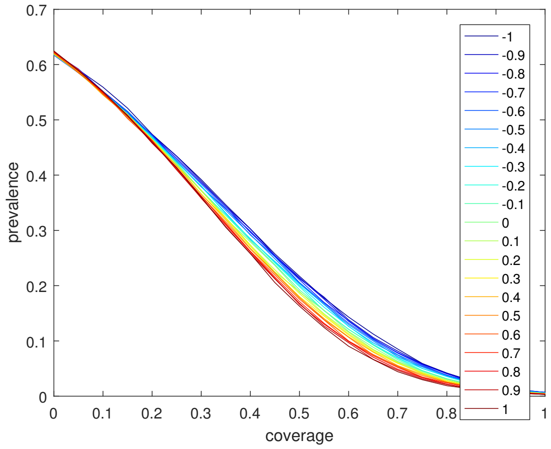

(b) SIS end prevalence, $\beta = 0.8$

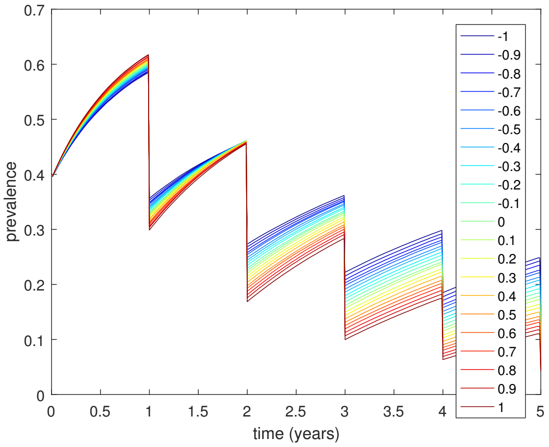

(c) Helminth simulation timecourses, $\beta = 0.25$

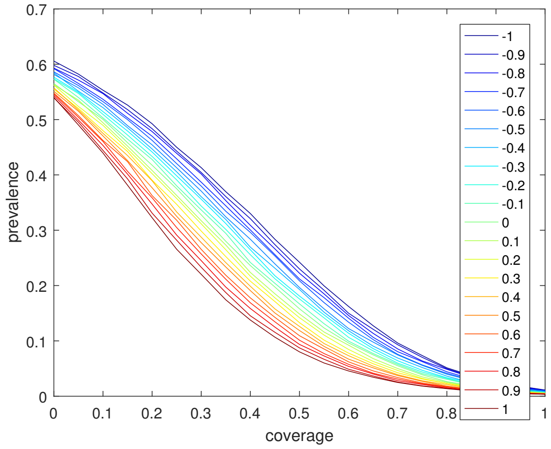

(d) Helminth end prevalence, $\beta = 0.25$

Figure 1: SIS and helminth simulations investigating the effect of correlations between the risk of infection and the probability of receiving treatment. Figures show: (a) and (c) prevalence in the population during a treatment campaign; (b) and (d) prevalence after 5 years. In each plot the lines represent different values of the correlation between infection risk and probabiltiy of receiving treatment, from negative (blue) to positive (red). SIS plots use an infectivity of $\beta = 0.8$ while helminth SIS plots use an infectivity of $\beta = 0.25$, and all plots were averaged over 100 simulations.

Poularikas, A. D., 1998. Handbook of formulas and tables for signal processing. Vol. 13. CRC Press.
